# An Estimate of the Global Burden of Anthropogenic Ozone and Fine Particulate Matter on Premature Human Mortality Using Atmospheric Modeling

**DOI:** 10.1289/ehp.0901220

**Published:** 2010-04-09

**Authors:** Susan C. Anenberg, Larry W. Horowitz, Daniel Q. Tong, J. Jason West

**Affiliations:** 1 University of North Carolina at Chapel Hill, Chapel Hill, North Carolina, USA; 2 Geophysical Fluid Dynamics Laboratory, National Oceanic and Atmospheric Administration, Princeton, New Jersey, USA; 3 Science and Technology Corporation, Silver Spring, Maryland, USA

**Keywords:** air pollution, atmospheric chemistry model, health effects of air pollution, health impact analysis, ozone, particulate matter

## Abstract

**Background:**

Ground-level concentrations of ozone (O_3_) and fine particulate matter [≤ 2.5 μm in aerodynamic diameter (PM_2.5_)] have increased since preindustrial times in urban and rural regions and are associated with cardiovascular and respiratory mortality.

**Objectives:**

We estimated the global burden of mortality due to O_3_ and PM_2.5_ from anthropogenic emissions using global atmospheric chemical transport model simulations of preindustrial and present-day (2000) concentrations to derive exposure estimates.

**Methods:**

Attributable mortalities were estimated using health impact functions based on long-term relative risk estimates for O_3_ and PM_2.5_ from the epidemiology literature. Using simulated concentrations rather than previous methods based on measurements allows the inclusion of rural areas where measurements are often unavailable and avoids making assumptions for background air pollution.

**Results:**

Anthropogenic O_3_ was associated with an estimated 0.7 ± 0.3 million respiratory mortalities (6.3 ± 3.0 million years of life lost) annually. Anthropogenic PM_2.5_ was associated with 3.5 ± 0.9 million cardiopulmonary and 220,000 ± 80,000 lung cancer mortalities (30 ± 7.6 million years of life lost) annually. Mortality estimates were reduced approximately 30% when we assumed low-concentration thresholds of 33.3 ppb for O_3_ and 5.8 μg/m^3^ for PM_2.5_. These estimates were sensitive to concentration thresholds and concentration–mortality relationships, often by > 50%.

**Conclusions:**

Anthropogenic O_3_ and PM_2.5_ contribute substantially to global premature mortality. PM_2.5_ mortality estimates are about 50% higher than previous measurement-based estimates based on common assumptions, mainly because of methodologic differences. Specifically, we included rural populations, suggesting higher estimates; however, the coarse resolution of the global atmospheric model may underestimate urban PM_2.5_ exposures.

Ground-level ozone (O_3_) and fine particulate matter [≤ 2.5 μm in aerodynamic diameter (PM_2.5_)] have increased substantially since preindustrial times. Although O_3_ and PM_2.5_ concentrations have increased most in industrialized areas, observations show that background concentrations have also increased in remote regions (Akimoto 2003; Schultz et al. 2006; Staehelin et al. 2001; Vingarzan 2004; Volz and Kley 1988). O_3_ and PM_2.5_ are associated with negative health impacts, including premature mortality (e.g., Jerrett et al. 2009; Krewski et al. 2009). Cohen et al. (2004) estimated that about 800,000 annual premature deaths globally, or 1.2% of all deaths, are associated with urban outdoor PM_2.5_. This was considered an underestimate because it excludes O_3_ impacts and includes only urban areas for which econometric models trained with observations were used to predict concentrations.

We estimated the global burden of human mortality due to anthropogenic O_3_ and PM_2.5_ using a global atmospheric chemical transport model (CTM). Using an atmospheric CTM allows estimation of mortality where air quality measurements are sparse, particularly in developing nations. By simulating preindustrial concentrations, we also isolated mortality due to anthropogenic pollution and avoided making assumptions for background O_3_ and PM_2.5_ concentrations. Global CTMs have been used to estimate mortalities due to long-range transport of air pollution (Anenberg et al. 2009; Liu et al. 2009; West et al. 2009), future changes in emissions (West et al. 2006, 2007), or changes in one sector’s emissions (Corbett et al. 2007). CTMs have not been used previously to quantify the global burden of anthropogenic air pollution on human mortality.

## Materials and Methods

We calculated mortalities associated with anthropogenic air pollution using health impact functions that relate changes in pollutant concentrations to changes in mortality. We defined anthropogenic air pollution as the geographically distributed difference between present-day (2000) and preindustrial O_3_ and PM_2.5_ concentrations, as simulated by a global CTM. Health impact functions for both O_3_ and PM_2.5_ are based on a log-linear relationship between relative risk (RR) and concentrations defined by epidemiology studies (e.g., Jerrett et al. 2009; Krewski et al. 2009):


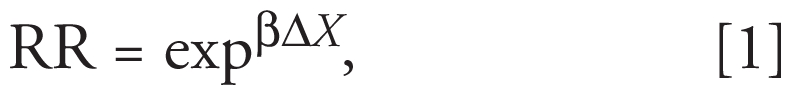


where β is the concentration–response factor (CRF; i.e., the estimated slope of the log-linear relation between concentration and mortality) and Δ*X* is the change in concentration. The fraction of the disease burden attributable to the risk factor, the attributable fraction (AF), was defined as


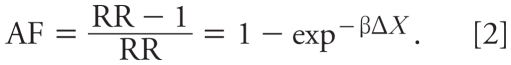


AF was multiplied by the baseline mortality rate (*y*_0_) and size of the exposed population (Pop) to yield an estimate of the excess mortalities attributable to air pollution (ΔMort):


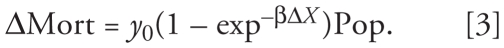


Disease survival time varies among populations, and we calculated years of life lost (YLL) associated with mortalities using the baseline YLL (YLL_0_) per death:


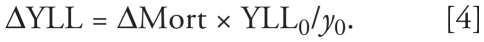


For O_3_, we based CRFs on the association between long-term O_3_ exposure and RR of death from respiratory disease found by Jerrett et al. (2009) in an American Cancer Society (ACS) cohort study of U.S. adults ≥ 30 years of age for 1977–2000. Although many daily time-series epidemiology studies demonstrate short-term O_3_-mortality impacts (e.g., Bell et al. 2004), Jerrett et al. (2009) provide the first clear evidence for long-term impacts. For the two-pollutant model that controlled for PM_2.5_, a 10-ppb increase in the seasonal (April–September) average daily 1-hr maximum O_3_ (concentration range, 33.3–104.0 ppb) was associated with a 4% [95% confidence interval (CI), 1.3–6.7%] increase in RR of death from respiratory disease.

For PM_2.5_, we used RRs from Krewski et al. (2009), which is the latest reanalysis of the ACS PM_2.5_ studies (e.g., Pope et al. 2002) and has the largest population of the available PM_2.5_ cohort studies (e.g., Hoek et al. 2002; Laden et al. 2006). We used RRs for 1999–2000 from the random-effects Cox model analysis that adjusted for 44 individual-level and seven ecological covariates. A 10-μg/m^3^ increase in PM_2.5_ (concentration range, 5.8–22.2 μg/m^3^) was associated with 6% (95% CI, 4–8%), 13% (95% CI, 10–16%), and 14% (95% CI, 6–23%) increases in total, cardiopulmonary, and lung cancer mortality. The linearity of the concentration–response function was also demonstrated up to 30 μg/m^3^ in the 1979–1983 analysis. Krewski et al. (2009) found that PM_2.5_ was associated most strongly with risk of death from ischemic heart disease, a subset of cardiopulmonary disease, and previous studies have found that controlling for O_3_ concentrations had little effect on the PM_2.5_–mortality relationships (Krewski et al. 2000). Compared with the relationships in an earlier expert elicitation (Roman et al. 2008), the total mortality RR in Krewski et al. (2009) is generally 3–14% lower per 10-μg/m^3^ increase with a tighter CI.

We assumed that these relationships found in the United States are valid globally. For O_3_, Jerrett et al. (2009) is the first study showing significant long-term impacts, but the short-term impact has been well documented in North America and Europe (e.g., Anderson et al. 2004; Bell et al. 2004). For PM_2.5_, similar long-term mortality results have been demonstrated in Europe (Hoek et al. 2002), but to date no PM_2.5_ cohort studies have been conducted in the developing world. Short-term O_3_ and PM_2.5_ studies in developing nations demonstrate relationships that are generally comparable with short-term studies in North America and Europe (Health Effects Institute International Scientific Oversight Committee 2004). Our assumption is further supported by evidence that concentration–mortality relationships do not vary significantly by sex, age, and race (Jerrett et al. 2009; Krewski et al. 2009; Zanobetti et al. 2000), although some sensitive populations may be at a higher risk. Because global causes of death differ from those in North America and Europe, we emphasized cause-specific mortality, which may have less error than estimates of all-cause mortality across different populations.

**Figure 1:**
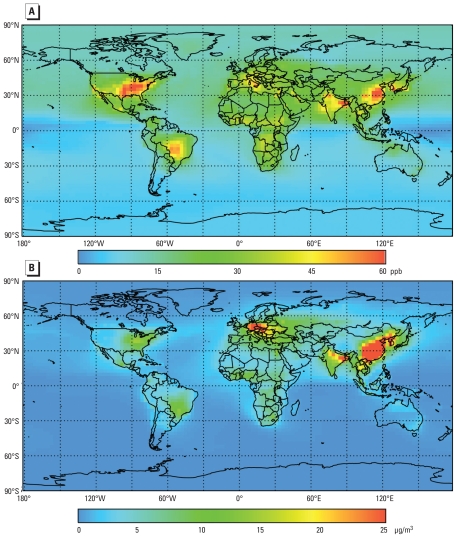
Estimated change (present minus preindustrial) in seasonal average (6-month) 1-hr daily maximum O_3_ concentrations (ppb; *A*) and annual average PM_2.5_ (μg/m^3^; *B*) from Horowitz (2006) simulations.

We used present-day (2000) and preindustrial O_3_ and PM_2.5_ concentrations (Figure 1) simulated by Horowitz (2006) using the Model of Ozone and Related Chemical Tracers, version 2 (MOZART-2; Horowitz et al. 2003). The preindustrial simulation, which corresponds to the 1860 simulation by Horowitz (2006), represents the “background” O_3_ and PM_2.5_ present in the absence of anthropogenic emissions, allowing us to isolate the anthropogenic contributions to concentrations and premature mortalities. MOZART-2 has a resolution of 2.8° latitude by 2.8° longitude with 34 vertical levels, and we used concentrations in the first vertical level as surface concentrations. Both simulations used the same meteorology from the National Center for Atmospheric Research Community Climate Model to isolate the impact of emission changes on concentration. We defined PM_2.5_ as all simulated sulfate (SO_4_), nitrate (NO_3_), ammonium, black carbon (BC), and primary organic carbon (OC). We excluded dust, sea salt, and secondary organic aerosols, which we assumed are unchanged from preindustrial to present. We multiplied OC mass by 1.4 to account for associated species other than carbon, and assumed all SO_4_ and NO_3_ exists as ammonium sulfate [(NH_4_)_2_SO_4_] and ammonium nitrate (NH_4_NO_3_), following Ginoux et al. (2006). For the preindustrial case, fossil fuel–burning emissions were set to zero and emissions from burning of biofuels, savannah, tropical forests, and agricultural waste were assumed to be 10% of 1990 values.

Consistent with the epidemiology studies, we used seasonal average 1-hr daily maximum concentrations for O_3_ and annual average concentrations for PM_2.5_. Because high O_3_ occurs during different months globally, for each grid cell, we found the consecutive 6-month period with the highest average of the simulated daily 1-hr maximum O_3_ concentrations, which we then used to calculate annual mortalities. Table 1 shows that the modeled global population-weighted seasonal average 1-hr daily maximum O_3_ increased by 37.1 ppb (from 19.6 ppb in 1860 to 56.7 ppb in 2000), using the present population, and the global population-weighted annual average PM_2.5_ increased by 15.0 μg/m^3^ (from 1.1 μg/m^3^ in 1860 to 16.1 μg/m^3^ in 2000). Globally, OC, BC, NO_3_, and SO_4_ are 62.3%, 6.3%, 0.3%, and 31.0% of total PM_2.5_ in 1860 and 45.6%, 9.1%, 4.9%, and 40.4% in 2000 [see Supplemental Material, Figure 1 (doi:10.1289/ehp.0901220)].

We compared modeled present-day surface O_3_ concentrations with data from the National Oceanic and Atmospheric Administration (NOAA) Earth Systems Research Laboratory Global Monitoring Division (NOAA 2008) monitoring network (mean bias = 2.5 ppb) for 11 remote locations around the world and from three nonurban networks: the Clean Air Status and Trends Network (U.S. EPA 2007) for the United States (mean bias = 2.9 ppb), the European Monitoring and Evaluation Programme (Convention on Long-Range Transboundary Air Pollution 2007) for Europe (mean bias = −0.2 ppb), and the Acid Deposition Monitoring Network in East Asia (EANET 2007) for Japan (mean bias = 0.4 ppb) [see Supplemental Material, Figures 2–5 (doi:10.1289/ehp.0901220)]. Horowitz (2006) found that simulated preindustrial O_3_ concentrations overestimate reconstructed observations from the late 19th century by approximately 5–10 ppb, with strong sensitivity to assumed biomass burning. Modeled surface PM_2.5_ concentrations were compared with observations by Ginoux et al. (2006) and were generally found to be estimated within a factor of 2 in remote locations and at nonurban stations in Europe and the United States, with a tendency to be overestimated. These comparisons show that MOZART-2 simulates surface O_3_ and PM_2.5_ well for nonurban and remote measurements in the areas compared, and it was not apparent that corrections for bias were necessary. Although simulated concentrations were not systematically biased outside of urban regions, the coarse resolution used here (grid cell area = 9.9 × 10^4^, 8.6 × 10^4^, and 5.2 × 10^4^ km^2^ at 0°, 30°, and 60° latitude) may cause errors in mortality estimates, particularly in urban areas with strong population and concentration gradients.

We estimated global premature mortalities separately for O_3_ and PM_2.5_ by applying Equation 3 in each of the MOZART-2 surface grid cells, using the corresponding population and baseline mortality rates for each cell. To calculate mortality, we used the global 2006 population (Oak Ridge National Laboratory 2008) [see Supplemental Material, Figure 6 (doi:10.1289/ehp.0901220)], and, consistent with the ACS study population, we used the population fraction ≥ 30 years of age (Table 1), estimated in 14 world regions [World Health Organization (WHO) 2004] [see Supplemental Material, Figure 7 (doi:10.1289/ehp.0901220)]. We used baseline all-cause, cardiopulmonary, and lung cancer mortality rates for 14 world regions (WHO 2004) and 66 countries (WHO 2008a), back-calculating from regional rates where country-specific rates were unavailable [Table 1; see also Supplemental Material, Figures 8–11 (doi:10.1289/ehp.0901220)]. Country-specific mortality rates are broadly categorized with no cutoff at 30 years of age, and we used rates for the population ≥ 25 years of age, assuming that differences between the rates are insignificant. We used baseline YLL rates for the population ≥ 30 years of age in 14 world regions [global average = 7.89, 9.77, and 8.93 for cardiopulmonary disease, respiratory disease, and lung cancer; see Supplemental Material, Table 1 (doi:10.1289/ehp.0901220)], assuming a 3% discount rate and nonuniform age weighting, giving less weight to years lived at older ages (WHO 2008b). We gridded baseline mortality rates, baseline YLL, and the fraction of the population ≥ 30 years of age to the MOZART-2 grid, and for grid cells overlapping multiple countries, we calculated area-weighted averages using a geographic information system program.

We present results as means ± 1 SD, calculating uncertainty from 500 Monte Carlo simulations that randomly sampled from normal distributions of the CRF, as reported by the epidemiology studies, and modeled present-day concentrations (SD = 25% of simulated value). Although the epidemiology literature provides little evidence for low-concentration thresholds (LCTs) or high-concentration thresholds (HCTs) for either O_3_ or PM_2.5_ (Jerrett et al. 2009; Krewski et al. 2009; Schwartz and Zanobetti 2000), mortality relationships beyond measured concentrations are unknown. Therefore, we estimated mortalities with and without assuming LCTs below which O_3_ and PM_2.5_ are assumed to have no effect on mortality. For O_3_, we applied an LCT of 33.3 ppb, the lowest measured level in Jerrett et al. (2009). When applied, this threshold replaced the natural background everywhere except in some grid cells in Asia and South America, where preindustrial concentrations exceeded the threshold (Table 1). We also examined an LCT of 56 ppb, which Jerrett et al. (2009) found to be close to statistical significance at an α-level of 5% (*p* = 0.0600). The 56-ppb threshold exceeded preindustrial concentrations in all cells. Because no grid cells exceeded the highest measured level (104.0 ppb) in Jerrett et al. (2009), we did not apply an HCT for O_3_. For PM_2.5_, we applied an LCT of 5.8 μg/m^3^, the lowest measured level in Krewski et al. (2009), which exceeded preindustrial concentrations in all grid cells (Table 1), effectively replacing the natural background. Some grid cells in Europe and Asia exceeded the highest measured level (30.0 μg/m^3^) in Krewski et al. (2009), and we examined HCTs of 30 μg/m^3^ and 50 μg/m^3^ in the sensitivity analysis. These thresholds applied only to our definition of PM_2.5_ and would be affected by including dust, sea salt, and secondary organic aerosols.

## Results

**Figure 2:**
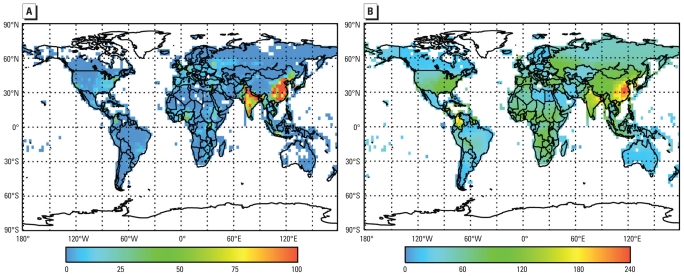
Estimated annual premature mortalities attributed to anthropogenic O_3_ when no upper or lower concentration threshold is assumed, for respiratory mortalities per 1,000 km^2^ (*A*) and rate of respiratory mortalities per 10^6^ people (*B*).

With no upper or lower concentration threshold, anthropogenic O_3_ was estimated to result in about 0.7 ± 0.3 million respiratory mortalities annually worldwide (Table 2), corresponding to 6.3 ± 3.0 million YLL (Table 3). Estimated global respiratory mortalities were reduced by approximately 33% when we assumed an LCT of 33.3 ppb, the lowest measured level in Jerrett et al. (2009). Regardless of threshold assumption, > 75% of O_3_ mortalities were estimated to occur in Asia, which is densely populated and highly polluted, whereas only approximately 5% occurred in North America. Estimated excess O_3_ mortalities were densest in highly populated areas but were distributed more evenly across the globe when divided (normalized) by population size (Figure 2).

**Figure 3:**
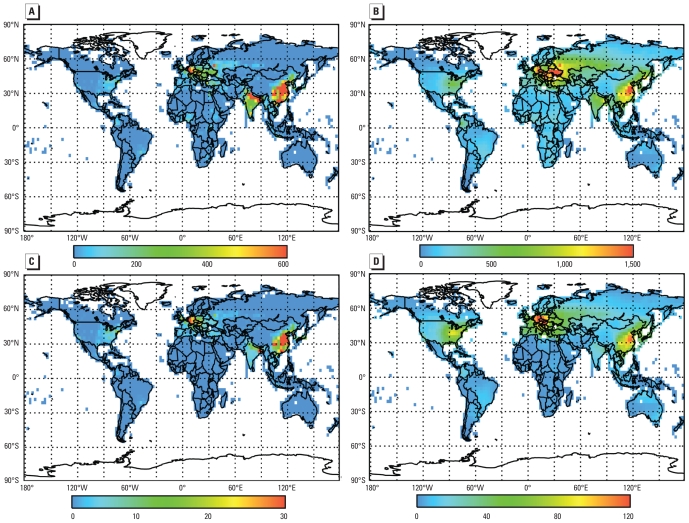
Estimated annual premature mortalities attributed to anthropogenic PM_2.5_ when no upper or lower concentration threshold is assumed, for cardiopulmonary mortalities per 1,000 km^2^ (*A*), rate of cardiopulmonary mortalities per 10^6^ people (*B*), lung cancer mortalities per 1,000 km^2^ (*C*), and rate of lung cancer mortalities per 10^6^ people (*D*).

Assuming no upper or lower concentration threshold, we estimated that exposure to anthropogenic PM_2.5_ results in 3.5 ± 0.9 million cardiopulmonary mortalities and 220,000 ± 80,000 lung cancer mortalities annually (Table 2), corresponding to 28 ± 6.8 and 2.2 ± 0.8 million YLL (Table 3). With an LCT of 5.8 μg/m^3^, estimated cardiopulmonary and lung cancer mortalities decreased by approximately 28%. Regardless of threshold, about 75% of excess mortalities occurred in Asia because of high PM_2.5_ concentrations and dense population, followed by Europe (17%). As for O_3_, estimated PM_2.5_ mortalities were densest in highly populated areas but more localized because of the shorter atmospheric lifetime of PM_2.5_ compared with O_3_ (Figures 1, 3). The highest estimated mortalities per million people were in Europe, East Asia, and the eastern United States (Figure 3B,D), owing to large baseline cardiopulmonary and lung cancer mortality rates and high PM_2.5_ concentrations.

Applying an LCT of 25 ppb for O_3_ resulted in approximately 14% fewer estimated respiratory mortalities than when assuming no upper or lower threshold (Table 4). With CRFs from the single-pollutant model in Jerrett et al. (2009), which did not control for PM_2.5_, O_3_-mortality estimates were approximately 25% lower, corresponding to the relative magnitudes of the CRFs. Applying the 56-ppb LCT from the threshold model reduced mortality estimates by approximately 75%. For PM_2.5_, RRs from Krewski et al. (2009) are similar to the 1979–1983 and 1999–2000 average all-cause and lung cancer RRs from Pope et al. (2002) but are approximately 40% higher for cardiopulmonary mortality, thus causing a corresponding increase in our estimates when applied [Table 5; see also Supplemental Material, Table 2 (doi:10.1289/ehp.0901220)]. Using RRs from Laden et al. (2006)—an extended reanalysis of the Harvard Six Cities cohort study that found significantly higher RRs than did Krewski et al. (2009)—increased estimated cardiopulmonary and lung cancer mortalities by approximately 30% and 50%, respectively. With no LCT, applying HCTs of 30 μg/m^3^ and 50 μg/m^3^ decreased estimated mortalities by approximately 10% and 1%, with larger decreases estimated for Europe and Asia, where some modeled concentrations exceeded the upper threshold values.

## Discussion and Conclusions

We estimated the global burden of mortality due to anthropogenic O_3_ and PM_2.5_ using a global atmospheric CTM and health impact functions. Anthropogenic O_3_ was associated with about 0.7 ± 0.3 million respiratory mortalities (1.1% ± 0.5% of all mortalities) and 6.3 ± 3.0 million YLL annually when we assumed no upper or lower concentration threshold. Anthropogenic PM_2.5_ was associated with about 3.5 ± 0.9 million cardiopulmonary (5.6% ± 1.4% of all mortalities) and 220,000 ± 80,000 lung cancer mortalities (0.4% ± 0.1% of all mortalities) annually when we assumed no threshold, corresponding to 30 ± 7.6 million YLL. Global mortalities were reduced by approximately 30% when we assumed LCTs of 33.3 ppb for O_3_ and 5.8 μg/m^3^ for PM_2.5_, the lowest measured levels in Jerrett et al. (2009) and Krewski et al. (2009). Estimated excess mortalities were densest in highly populated areas but also occurred in rural areas that have been affected by the increased regional or global background of air pollution since preindustrial times. These estimates based only on cardiopulmonary and lung cancer mortality may be conservative because O_3_ and PM_2.5_ may also affect other causes of mortality. In addition, to be consistent with the ACS study population, we included only the population ≥ 30 years of age, but evidence suggests that O_3_ and PM_2.5_ affect health negatively for all ages, including the very young [see U.S. Environmental Protection Agency (EPA) 2008 and references therein].

Estimated PM_2.5_ mortalities were five times O_3_ mortalities, suggesting that PM_2.5_ is the dominant contributor to the global health burden of outdoor air pollution. To minimize double counting of mortalities, we applied long-term RRs for O_3_ and PM_2.5_ based on the same ACS cohort. PM_2.5_ RRs have been shown to be independent from O_3_ concentrations (Krewski et al. 2000), and we used O_3_ RRs from Jerrett et al. (2009) that controlled for PM_2.5_. Furthermore, Jerrett et al. (2009) and Krewski et al. (2009) reported that PM_2.5_-related mortality was dominated by cardiovascular mortality, whereas O_3_ was primarily associated with respiratory mortality. The independence of the exposure–response relationships and the difference in dominant biological mechanisms of mortality for each pollutant imply that double counting is unlikely to be significant. If these implications are correct, O_3_ and PM_2.5_ mortalities may be summed together to yield total mortalities; otherwise, summing the results would overestimate total mortalities.

Mortality estimates were sensitive to concentration thresholds and concentration–mortality relationships, often changing by > 50% of the estimated value under different assumptions. We assumed that the CRFs found by epidemiology studies conducted in North America apply globally, despite differences in health status, lifestyle, age structure, and medical care, and emphasize cause-specific mortality. The CRFs used here could also be subject to confounders, including temperature (Jerrett et al. 2009). Although some evidence suggests differential toxicity of PM_2.5_ components (Franklin et al. 2008; Ostro et al. 2010), we assumed that all PM_2.5_ species exert effects similar to aggregated PM_2.5_, despite differences in PM_2.5_ composition throughout the world. These assumptions, although necessary because of limited data, may have substantial impacts on the results.

Using the same assumptions for CRFs (Pope et al. 2002 for 1979–1983) and LCTs [7.5 μg/m^3^, applied to total PM_2.5_ in Cohen et al. (2004), but here only to the species in our definition of PM_2.5_], our mortality estimates for urban and rural areas (Table 5) were approximately 50% higher than the previous estimate of 800,000 mortalities from urban air pollution reported by Cohen et al. (2004). The discrepancy results from two competing differences in methods. First, we included rural populations, which were excluded by Cohen et al. (2004). Because the urban population in Cohen et al. (2004) is approximately 30% of the total global population, and air pollution has increased in rural regions, including rural populations suggests many more air pollution mortalities globally. Second, we used a coarse-resolution global CTM that spread emissions across large grid cells. Although rural O_3_ and PM_2.5_ were well simulated, the coarse resolution may suppress high urban PM_2.5_ concentrations, causing underestimates of PM_2.5_ mortalities. Compared with a previous U.S. estimate of 144,000 PM_2.5_ mortalities (all causes) found using a regional CTM (U.S. EPA 2009), our estimate of PM_2.5_ mortalities in North America is similar but slightly lower (by 2% for cardiopulmonary and lung cancer mortalities and 7% for all-cause mortalities). The coarse-resolution model may either overestimate or underestimate O_3_ pollution in urban areas because O_3_ precursors were diluted into a large volume. Previous studies have found that regional O_3_ can be overestimated by 13% at the resolution used here but that background concentrations are not greatly affected by resolution (Wild and Prather 2006). A finer-resolution model would capture urban populations and concentrations more accurately, but adequate resolution is not currently possible in global air quality models.

Despite these limitations, this study highlights regions where improvements to air quality may be particularly effective at reducing mortality related to air pollution. Previous estimates rank urban air pollution (PM_2.5_ only) as the 13th leading global mortality risk factor and third among environmental risks (Ezzati et al. 2004). Our results suggest a larger burden of disease due to outdoor air pollution than was previously estimated but should be compared with other risk factors only when all are updated consistently. Future estimates of the global burden of air pollution on mortality should strive to combine information from global and regional models with rural and urban concentrations from measurements. These estimates should also incorporate CRFs from new studies on O_3_– and PM_2.5_–mortality relationships that examine individual PM_2.5_ components, that are conducted in different parts of the world, that include populations of all ages, and that resolve relationships at low and high concentrations. In the future, global economic development will likely shift the disease burden from infectious disease and malnutrition to chronic conditions, which are more strongly affected by air pollution exposure. Although some countries have implemented policies to improve air quality, without further action the global burden of anthropogenic air pollution on mortality may be even larger in the future than is estimated for the present.

## Figures and Tables

**Table 1:** Population ≥ 30 years of age, average baseline mortality rates, and population-weighted average and range of the seasonal average (6-month) 1-hr daily maximum O_3_ concentrations and annual average PM_2.5_ concentrations from MOZART-2 simulations of preindustrial (1860) and present-day (2000) levels.

	Pop ≥ 30^b^ (billions)	Baseline mortality rates (%/year)^c^			O_3_ (ppb)^a^				PM_2.5_ (μg/m^3^)^a^			
					1860		2000		1860		2000	
		Respiratory	CP	LC	Average	Range	Average	Range	Average	Range	Average	Range
Africa	0.28	0.206	0.739	0.011	23.32	11.4–31.9	54.46	20.2–71.5	0.92	0.28–3.19	7.50	0.50–13.9
North America	0.27	0.081	0.502	0.071	21.42	12.5–32.3	59.75	27.0–89.3	1.50	0.14–4.65	8.44	0.31–16.6
Europe	0.44	0.127	1.22	0.056	18.26	15.2–27.5	48.92	32.3–74.3	0.93	0.11–2.96	14.77	0.40–39.0
Asia	1.8	0.171	0.746	0.037	18.91	6.15–35.9	59.64	10.8–83.7	1.19	0.23–3.06	20.41	0.34–55.9
South America	0.15	0.121	0.515	0.025	18.44	12.2–35.8	44.59	22.3–90.3	1.00	0.33–3.89	6.35	0.40–13.9
Oceania	0.02	0.074	0.346	0.035	13.37	3.74–22.8	26.75	6.41–44.4	0.96	0.22–2.27	2.59	0.25–5.01
World	2.9	0.134	0.754	0.042	19.61	3.74–35.9	56.70	6.41–90.3	1.13	0.11–3.89	16.11	0.25–55.9

Abbreviations: CP, cardiopulmonary; LC, lung cancer; Pop, population. Data are average and range for the highest and lowest individual grid cells.

a Simulated by Horowitz (2006).

b Population ≥ 30 years of age for the year 2006 from the LandScan database (Oak Ridge National Laboratory 2008).

c Baseline mortality rates are country specific for the latest year after 2000 with data available (WHO 2008a). Where country-specific rates after the year 2000 were not available, we back-calculated country-specific rates from regional rates for the year 2002.

**Table 2:** Estimated annual mortalities ± 1 SD due to anthropogenic O_3_ and PM_2.5_, assuming natural background only or LCTs (33.3 ppb for O_3_ and 5.8 μg/m^3^ for PM_2.5_) (× 1,000).

	O_3_ respiratory		PM_2.5_ cardiopulmonary		PM_2.5_ lung cancer	
	Background	Threshold	Background	Threshold	Background	Threshold
Africa	63 ± 34	45 ± 30	154 ± 44	52 ± 33	3 ± 1	1 ± 1
North America	35 ± 17	25 ± 15	124 ± 37	65 ± 30	17 ± 7	10 ± 5
Europe	41 ± 21	23 ± 17	586 ± 149	383 ± 143	47 ± 17	31 ± 14
Asia	543 ± 253	370 ± 220	2,584 ± 618	1,991 ± 603	152 ± 53	122 ± 47
South America	18 ± 9	8 ± 6	48 ± 15	16 ± 9	2 ± 1	1 ± 1
Oceania	1 ± 1	0 ± 0	2 ± 1	0 ± 0	0 ± 0	0 ± 0
World	700 ± 335	470 ± 288	3,499 ± 864	2,506 ± 816	222 ± 80	164 ± 68

SDs reflect uncertainty in the CRF and simulated present-day concentrations (SD = 25% of simulated concentration).

**Table 3:** Estimated annual YLL ± 1 SD due to anthropogenic O_3_ and PM_2.5_, assuming the natural background or LCTs (33.3 ppb for O_3_ and 5.8 μg/m^3^ for PM_2.5_) (× 1,000).

	O_3_ respiratory		PM_2.5_ cardiopulmonary		PM_2.5_ lung cancer	
	Background	Threshold	Background	Threshold	Background	Threshold
Africa	901 ± 486	644 ± 429	1,694 ± 484	572 ± 363	40 ± 13	13 ± 13
North America	285 ± 138	203 ± 122	804 ± 240	421 ± 194	152 ± 62	89 ± 45
Europe	243 ± 125	136 ± 101	4,336 ± 1,103	2,834 ± 1,058	472 ± 171	311 ± 141
Asia	4,322 ± 2,014	2,945 ± 1,751	20,620 ± 4,932	15,888 ± 4,812	1,594 ± 556	1,280 ± 493
South America	137 ± 68	61 ± 46	365 ± 114	122 ± 68	19 ± 10	10 ± 10
Oceania	7 ± 7	0 ± 0	11 ± 6	0 ± 0	0 ± 0	0 ± 0
World	6,251 ± 2,992	4,197 ± 2,572	27,607 ± 6,817	19,772 ± 6,438	2,169 ± 782	1,602 ± 664

SDs reflect uncertainty in the CRF and simulated present-day concentrations (SD = 25% of simulated concentration).

**Table 4:** Estimated annual global O_3_ mortalities (mean ± 1 SD) using CRFs from the multipollutant model (in which PM_2.5_ was controlled) and single-pollutant model in Jerrett et al. (2009), and LCTs (×1,000).

	Cardiopulmonary	Respiratory
Multipollutant model	—	700 ± 335
LCT = 25 ppb	—	605 ± 317 (−13.6%)
LCT = 33.3 ppb	—	470 ± 288 (−32.9%)
Single-pollutant model	1,076 ± 493	524 ± 252 (−25.1%)
LCT = 25 ppb	925 ± 467	452 ± 238 (−35.4%)
LCT = 33.3 ppb	705 ± 423	350 ± 215 (−50.0%)
Threshold model^a^		
LCT = 56 ppb	—	178 ± 187 (−74.6%)

Data in parentheses are percentage change from estimates assuming CRFs from Jerrett et al. (2009) multipollutant model with no LCT (top row). Uncertainty is from the CRF and simulated present-day concentrations (SD = 25% of simulated concentration).

a Calculated using the CRF (0.00432 ppb^−1^) and corresponding standard error (0.00121 ppb^−1^) for respiratory mortality when a threshold of 56 ppb is included in the O_3_-mortality model (Jerrett et al. 2009). Although Jerrett et al. (2009) found that no threshold model was clearly a better fit to the data than a linear representation of the overall O_3_–mortality association, a threshold of 56 ppb was close to statistical significance (*p* = 0.06).

**Table 5:** Estimated annual global PM_2.5_ mortalities (mean ± 1 SD) using alternative CRFs with and without LCTs and HCTs (×1,000).

	Mortality		
	All causes	Cardiopulmonary	Lung cancer
Krewski et al. (2009)	3,381 ± 986	3,499 ± 864	222 ± 80
LCT = 5.8 μg/m^3^	2,378 ± 876 (−29.7%)	2,506 ± 816 (−28.4%)	164 ± 68 (−26.1%)
LCT = 7.5 μg/m^3^	2,077 ± 822 (−38.6%)	2,201 ± 780 (−37.1%)	146 ± 64 (−34.2%)
HCT = 30 μg/m^3^	3,059 ± 774 (−9.5%)	3,205 ± 676 (−8.4%)	201 ± 68 (−9.5%)
HCT = 50 μg/m^3^	3,338 ± 940 (−1.3%)	3,464 ± 826 (−1.0%)	219 ± 78 (−1.4%)
Pope et al. (2002), 1979–1983^a^	2,333 ± 1,196 (−31.0%)	1,800 ± 742 (−48.6%)	139 ± 72 (−37.4%)
Laden et al. (2006)^b^	7,714 ± 2,736 (+128.2%)	4,549 ± 1,439 (+30.0%)	336 ± 198 (+51.4%)

Data in parentheses are percentage change from estimates assuming CRFs from Krewski et al. (2009) and no LCT or HCT (top row). Uncertainty is from the CRF and simulated present-day concentrations (SD = 25% of simulated concentration).

a Pope et al. (2002) reported RR estimates for two time periods (1979–1983 and 1999–2000) and for the integrated average of both. The RR estimates for 1979–1983 were more conservative than those from 1999–2000 and the integrated average. See Supplemental Material, Table 2 (doi:10.1289/ehp.0901220), for results from the average of both time periods and with concentration thresholds.

b Laden et al. (2006) extended the follow-up of the Harvard Six Cities adult cohort study for 8 years and found significantly higher RR estimates for overall mortality than did the original study or Krewski et al. (2009). See Supplemental Material, Table 2 (doi:10.1289/ehp.0901220), for results with concentration thresholds.
